# “Well, I Signed Up to Be a Soldier; I Have Been Trained and Equipped Well”: Exploring Healthcare Workers’ Experiences during COVID-19 Organizational Changes in Singapore, from the First Wave to the Path towards Endemicity

**DOI:** 10.3390/ijerph19042477

**Published:** 2022-02-21

**Authors:** Celene Ting, Alyssa Yenyi Chan, Lai Gwen Chan, Zoe Jane-Lara Hildon

**Affiliations:** 1 Saw Swee Hock School of Public Health and National University Health System, National University of Singapore, Tahir Foundation Building, 12 Science Drive 2, Level 09-03J, Singapore 117549, Singapore; ephv295@nus.edu.sg (C.T.); alyssa.c@nus.edu.sg (A.Y.C.); 2 Department of Psychiatry, Tan Tock Seng Hospital, 11 Jalan Tan Tock Seng, Singapore 308433, Singapore; lai_gwen_chan@ttsh.com.sg; 3 National Centre for Infectious Diseases (NCID), Ministry of Health of Singapore, 16 Jln Tan Tock Seng, Singapore 308442, Singapore

**Keywords:** COVID-19, qualitative study, pandemic preparedness, healthcare workers’ morale, quality and safety, improved systems functioning

## Abstract

(1) Background: As COVID-19 transmission continues despite vaccination programs, healthcare workers (HCWs) face an ongoing pandemic response. We explore the effects of this on (1) Heartware, by which we refer to morale and commitment of HCWs; and identify how to improve (2) Hardware, or ways of enabling operational safety and functioning. (2) Methods: Qualitative e-diary entries were shared by HCWs during the early phases of the outbreak in Singapore from June to August 2020. Data were collected via an online survey of *n* = 3616 HCWs of all cadres. Nine institutions—restructured hospitals (*n* = 5), affiliated primary partners (*n* = 2) and hospices (*n* = 2)—participated. Applied thematic analysis was undertaken and organized according to Heartware and Hardware. Major themes are in *italics* (3) Results: *n* = 663 (18%) HCWs submitted a qualitative entry. Dominant themes undermining (1) Heartware consisted of *burnout from being overworked and emotional exhaustion* and at times feeling a *lack of appreciation or support at work*. The most common themes overriding morale breakers were *a stoic acceptance to fight, adjust and hold the line,* coupled with *motivation from engaging leadership and supportive colleagues*. The biggest barrier in (2) Hardware analysis related to *sub-optimal segregation strategies within wards* and designing *better protocols for case detection, triage, and admissions criteria*. Overall, the most cited enabler was the *timely and well-planned provision of Personal Protective Equipment (PPE) for front-liners*, though scope for scale-up was called for by those not considered frontline. Analysis maps internal organizational functioning to wider external public and policy-related narratives. (4) Conclusions: COVID-19 surges are becoming endemic rather than exceptional events. System elasticity needs to build on known pillars coupling improving safety and care delivery with improving HCW morale. Accordingly, a model capturing such facets of Adaptive Pandemic Response derived from our data analyses is described. HCW burnout must be urgently addressed, and health systems moved away from reactive “wartime” response configurations.

## 1. Introduction

### 1.1. Background

Concern over the well-being and resilience of healthcare workers [1,2,3] (HCWs) worldwide continues to rise with the emergence of new and more transmissible variants [4] of the COVID-19 virus. At the time of writing, several countries are facing new waves and new challenges to contain these ongoing surges as they hit the tertiary care health system [5]. The most affected are HCWs in health systems that were unprepared for the relentless, unpredictable rates of transmission, and an onslaught of challenges that while different in nature have not discriminated in their intensity between low- and high-income countries [6].

The current study explores e-diary qualitative data of HCWs in Singapore during the latter phases of the first wave (June–August 2020) to better understand how to bridge workforce morale and operational safety and functioning. We report our study according to the Standards for Reporting Qualitative Research (SRQR) [7], beginning by outlining the study background and problem formulation.

### 1.2. Morale and Commitment of Healthcare Workers

Data collected from hospital and frontline HCWs as the outbreak first unfolded in Singapore will help inform current and future intervention priorities. Recent studies show that high levels of fear, hopelessness and stress caused by working under the duress of the pandemic can lead to a myriad of adverse outcomes for hospital HCWs [8,9,10,11], for instance, affecting HCWs’ self-efficacy in care delivery [12] and, ultimately, impeding the operational functioning of tertiary care institutions.

In addition, aside from compromised psycho-emotional states [13,14], HCWs’ physical health functioning [15] can also be affected, contributing to impaired performance and/or absenteeism. For example, cumulative stress combined with lack of hope among HCWs has been demonstrated to affect perceptions of incidences of medical errors in care delivery [16]. At the organizational level, work-related stress due to overwork, trauma and perceived inequitable compensation will impact turnover and intentions to stay [17].

### 1.3. Operational Safety and Functioning 

Pandemic preparedness has been at the center of Singapore’s National Infectious Disease (ID) strategy since the SARS-CoV outbreak of 2003. Recent planning analysis has emphasized strategies that account for different aspects of capacity for crowding as well as the capability for treating highly complex emerging ID threats [18,19]. Accordingly, threat scenarios are described as unfolding in different ways.

The highest level of surge threat for a hospital to be overwhelmed was described as the point where the world as we know it “ends as it extends” [19]. Such a scenario occurs when the system is overburdened *both* by crowding and the complexity or novelty of the pathogen (e.g., Spanish Flu, Ebola, and COVID-19). This is the situation currently faced by many health systems globally and an eventuality Singapore has long planned for, recognizing the need to build flexibility into the system [18,20].

### 1.4. Problem Formulation 

The Singapore tertiary system therefore presents both an opportunity for transferable learning [21] and for further study to identify lingering and unforeseen system challenges. In line with this, we focus the current analysis on staff perceptions of both *Heartware* and *Hardware* using these constructs to frame analyses. Lim et al. (2020) [22] use the term Heartware to describe long-term psychological impacts which impede performance and jeopardize HCW and patient safety; herein, we refer to it as primarily reflective of factors affecting staff morale and commitment. As for Hardware, Lim et al. go on to define it as referring to environmental and infection controls, such as suitable use of Personal Protective Equipment (PPE) [22]; we more broadly refer to the term as capturing ways of enabling operational safety and functioning.

## 2. Research Questions 

Accordingly, the study research questions are as follows; during the first wave of the pandemic in Singapore:

1. How was morale experienced by HCWs in terms of breaking as well as boosting engagement and commitment?
2. What barriers and enablers were experienced that affected operational safety and functioning?

## 3. Methods

### 3.1. Qualitative Approach and Research Paradigm 

We undertook a qualitative analysis of e-dairy data, which were collected using an online version of the ‘mailbox technique’ [23]. Due to the difficulty in accessing HCWs during a time of heavy infection control restrictions and people being burdened with long shift work and overtime, we opted to invite HWCs to share what they felt and observed via journaling, sharing comments and/or uploading audio and photos. The current e-diary approach is connected to organizational psychology research [24]. In particular, it addresses the problem of how to reach unseen and unheard worlds that otherwise could be out of reach. It can often combine several types of multimedia data, and/or be triangulated across methods. Triangulation is used when wanting to interrogate the same question using multiple data sources. The data collection itself is also underpinned by principles of catharsis, intimate sharing and/or self-reflection that can aid during times of great stress or upheaval.

### 3.2. Researcher Team Composition and Reflexivity

The data were collected via a survey method designed by a psychiatrist and extended team (L.G.C.) working in the ID and tertiary care Singapore landscape. Qualitative data were extracted and analyzed by a cross-disciplinary team trained in thematic analysis (Z.J.L.H., C.T. and A.Y.C.) with backgrounds in Sociology, Psychology, Communication and Healthcare Administration. The process was guided by a senior qualitative and mixed-methods researcher (Z.J.L.H.) familiar with the historical ID outbreaks and pandemics preparedness strategy in Singapore.

### 3.3. Context

Nine healthcare institutions, consisting of restructured hospitals (*n* = 5), as well as affiliated primary care polyclinics (*n* = 2), and intermediate and long-term care (ILTC) hospices (*n* = 2), participated in the anonymous, online survey that was disseminated from mid-July to mid-August 2020. This was approximately one month after a two-month lockdown termed the ‘Circuit Breaker’. During that period, the health system faced a large outbreak mostly contained within migrant worker dormitories [25] and a number of small community clusters. During this period, only two people were reported deceased from COVID-19 [26]. Stringent quarantine and contact tracing measures were in place.

### 3.4. Sampling Strategy 

All cadres from frontline clinical teams to allied health and administrative staff and pharmacists, etc., were invited to participate anonymously via institutional email. 

### 3.5. Ethical Issues

Ethical approval for this study was obtained from the National Healthcare Group Domain Specific Review Board (DSRB), Singapore (Reference N^o^. Is 2020/00385), and all participants gave e-consent to participate in this study. Institutional approvals were obtained from participating organizations. Nevertheless, in the interest of confidentiality and to preserve anonymity, names of participating institutions are blinded; in addition, only cadres and minimal demographic content are tagged to quotes.

### 3.6. Data Collection Methods 

The qualitative open-ended component of the survey forms the focus of our analysis. Participation was voluntary and no reimbursements were offered for participation. Participants were informed that they could choose not to participate or complete the questionnaire in full anonymity. Participants were asked through an open-ended response section/question to share any impressions they had of working in their current roles since the outbreak—this could take the form of a journal or diary entry, photo, poem or drawing, Supplementary Box S1 for questionnaire format. Comments on suggestions to improve organizational functionality were also invited.

### 3.7. Data Collection Instruments and Technologies 

Data collection was administered through Qualtrics, a secure online platform that allowed participants to craft diary entries, save and return prior to submission and upload multimedia files. The survey link was blasted through staff institutional email of participating institutions, and the quantitative component is reported elsewhere [27].

### 3.8. Units of Study

The overall response rate to the qualitative component of the survey was 18% (*n* = 663 HCW of 3616 eligible participants). The characteristics of respondents are summarized below.

### 3.9. Data Processing 

Data were extracted from the main survey dataset and organized by institution. Key defining variables are also retained alongside the journaling and other e-dairy entries in a matrix format. Media images and audio were described—for example, pictures of “PPE marks imprinted on skin after removal of face coverings” and “a group of nurses smiling, doing facials together in shared accommodation.” Audio summaries as well as comments on how to improve things were also extracted into the grids. All were collated in Microsoft Word and matrices were then imported into Atlas.ti for coding by the team.

### 3.10. Data Analysis 

Applied thematic analysis was undertaken [28]. Steps included data familiarization, creating a shared coding framework and codebook and agreeing on emergent themes and sub-themes. Following Guest et al. (2012)’s assertion that applied thematic analysis can be used to “build theoretical models or to find solutions to real world problems”, p. 17 [28], this was the final step in analysis.

The coding framework was organized by elements that broke or boosted morale as well as perceptions of infrastructural barriers and enablers affecting operational safety and functioning (internal to institutions) and contrasted to views of broader policy and contextual factors (external to institutions). Aspects relating to mental health, coping and individual rather than organizational functioning were identified and segmented for separate, future analyses.

In the current analysis, once themes were identified, they were further grouped into observed “manifest” outcomes to the broader “latent” ones that explain them. The process is an extension of thematic coding and relies on the coder to identify the type and sequence of outcomes and to highlight observed relationships or system linkages.

### 3.11. Techniques to Enhance Trustworthiness 

The team met regularly to identify and agree on broad themes and supporting sub-themes, across the institutions allocated to each coder. Saturation was judged to have been reached since all coders were able to collapse the identified themes, with no new themes emerging in the final stages of coding. 

According to Guest et al. (2012), applied thematic analysis shares both interpretivist and positivist underpinnings and, thus, in certain instances, recording the size or recurrence of codes can be useful [28]. The method is intended to inform solutions to real-world problems with practical data. Therefore, we have retained and recorded the size of codes to denote common, recurring points and experiences. Though we recognize there is some controversy surrounding sharing codes in this way, we do so in aid of transparency and acknowledgement of the way the data was compiled (via a survey platform). By no means do we infer any statistical meaning from these top-line recordings, nor that smaller or minority themes are necessarily less important. 

Themes are recorded in *italics* together with number of occurrences and narrated alongside supporting sub-themes.

## 4. Results

Eighteen percent (*n* = 663 participants) submitted an included qualitative entry; see Supplementary Table S1 for breakdown of responders vs. non-responders. 

Nine healthcare institutions participated, with more and less handling of COVID-19 patients; see breakdown in Supplementary Table S2. 

Somewhat more non-locals (57.5%) rather than locals responded, just over half the respondents were married (51.7%) or lived with family (55.2%), and many more women (76.5%) and nurses (49.3%) responded. Most were aged 31 to 50 years old (56.4%). Seventeen percent reported daily exposure to COVID-19 patients. Results pertaining to each of the study research questions are now reported in turn, and illustrative quotes are listed in Table 1, Table 2, Table 3 and Table 4. 

### 4.1. How Was Morale Experienced by Healthcare Workers in Terms of Breaking as well as Boosting Engagement and Commitment? 

#### 4.1.1. Heartware: Morale Breakers

The most commonly shared theme undermining Heartware was primarily the cumulative *burnout from being overworked and emotional exhaustion* (*n* = 94). The general feeling of too much to do in too little time despite all hands (and hearts) on deck was vividly expressed, for instance, relating to juggling new administrative procedures and protocols on top of clinical work. 

Another facet of burnout was compassion fatigue, resulting from assimilating the emotional exhaustion of dealing with highly emotive patient and family interactions, or feeling conflicted in duty of care. Some HCWs said they suffered sleepless nights due to feelings of guilt and helplessness when interacting with patients and families whom they had forged close bonds with. 

Not being able to take adequate leave and make time for self-care greatly contributed to experiences of burnout in certain institutions. The disparity in blocking leave allowance and manpower allocation among departments within and across healthcare institutions was a clear demoralizer. 

*Lack of appreciation or support at work* (*n* = 56) was also a dominant theme, deeply affecting morale. From the management side, HCWs spoke about lacking support to deal with push–pull factors such as lack of leave and childcare issues affecting work–life balance. Even when feedback to improve work conditions were sought, this was not seen to lead to changes. 

The broader theme of feeling unappreciated emerged across genders, professional cadres and institutions, but was especially voiced by the younger HCWs. Female nurses in particular shared that feeling this way stemmed from long-standing hierarchical disparities, which were exacerbated under COVID-19 working conditions. Similarly, cadres or institutions not considered frontline “enough” expressed feeling like they were fighting their share of the COVID-19 battle in the shadows. 

In addition, the public’s appreciation was seen by some as meaningless if management was not also pushing government to step up incentives. Moreover, difficult patients aggravated feeling unappreciated. In the worst cases, the above-described experiences can be interpreted as conflating, leading to more intense feelings of *disengagement* (*n* = 12), characterized by a sense of betrayal at not being heard and related moral injury, and feeling conflicted about duty of care, ultimately, causing healthcare workers to consider leaving. Lastly, in very few instances, *workplace stigma* (*n* = 5), leading to a sense of disunity, was described by staff with direct or possible exposure to COVID-19.

Some also talked about *feeling stigmatized or discriminated against by the wider community* (n = 17), meaning that a sense of disunity was further emphasized either because healthcare workers are assumed to have or have declared having close contact with COVID-19 patients. More broadly, there were *calls for better pay and provisions for healthcare workers* (n = 16). This included, national policy catering for better provision for healthcare workers, e.g., better pay and working conditions, certain privileges such as green lanes for shopping and enabling those seeking to travel, as well as more equity between foreign and local healthcare workers. Others described *frustration about public apathy* (*n* = 7), caused by those observed not to be following rules and guidelines and/or being ignorant about the importance of preventative habits.

**Table 1:** Summary of themes and sub-themes with illustrative quotes for breaking morale, hindering engagement and commitment of healthcare workers—Heartware: morale breakers.

Themes		Supporting Sub-Themes with Illustrative Quotes
Internal—Institutional	*Burnout from being overworked and emotional exhaustion (n = 94)*	Too much to do in too little time *“Firefighting Administratively everyday–it’s difficult. I much rather just do clinical work. There were periods when I just wanna dump the bureaucracy behind and volunteer my services.”* Middle-aged female, local Doctor, #133, Hospital B Compassion fatigue *“Our dying patients were denied visitors altogether, despite being 2x COVID swabs negative. We appealed to the management to allow at least one visitor on compassionate grounds. Nurses and Doctors are at the peak of “compassion fatigue”. Some cried after shifts, others started finding it challenging to face [the relatives of] those patients we lost when they begged to see the deceased one last time before he/she is sent to the mortuary…ungodly moments where I hardened my heart or refused to respond. It’s take more than just praying on bended knees, and I struggled.”* Middle-aged female, local Nurse, #131, Hospital B Not being able to take adequate leave and make time for self-care *“While [some] colleagues […] continue to be able to clear leave, our staff are forced to take maximum 3 days leave a month (after having our leave suspended for more than 2 months), and in some departments…forced to even ballot for leave days or take enforced random days of leave which disrupt work. We are not afraid of hard work but would like to at least be treated fairly in terms of leave policies and enforced breaks.”* Younger male, local Doctor, #25, Hospital D
	*Lack of appreciation or support at work (n = 54)*	Lacking support to deal with push–pull factors such as lack of leave and childcare issues affecting work–life balance *“Multiple feedback sessions […] seem to have left most of our concerns unaddressed, just told to embrace public servanthood and personal sacrifice for the greater good with no real means of bettering our work life balance.”* Younger male, local Doctor, #25, Hospital D Hierarchical disparities, exacerbated under COVID-19 working conditions *“And one more thing that still bothers me is, NURSES ARE THE BACKBONE OF THE HEALTHCARE and yet, we are often not paid/ respected as much as doctors.”* Younger female, local Nurse, #16, Hospital E Cadres or institutions not considered frontline “enough” expressed feeling like they are fighting their share of the COVID-19 battle in the shadows *“We are definitely frontline healthcare workers but yet are often overlooked and underappreciated for the work we do.”* Younger male, local Pharmacist, #156, Hospital B *“The limelight was all [on the well-known Infectious Diseases institutions], minimal coverage of the daily struggles in a normal hospital setting…”* Middle-aged female, local Doctor, #133, Hospital B Public appreciation was seen by some as meaningless if management was not also pushing government to step up incentives *“[…] end it all. Stop clapping, singing calling us heroes. We need incentives and pay us for the hard work we did and the sacrifices we did, we are also human. We are tired.”* Younger male, local Nurse, #203, Hospital A Difficult patients *“Sometimes we felt the frustration for unnecessary complain handling e.g. [patient] refuse to wear mask and when we do our part we need to spend unnecessary time resolving the complain [sic], some complains [sic] are really a waste of time–we have to answer long complains because someone escalated the matter.”* Middle-aged female, local Nurse, #119. Hospital D
	*Disengagement (n = 12)*	Sense of betrayal at not being heard is related to moral injury, and feeling conflicted about duty of care, making healthcare workers consider leaving *“[This] COVID experience would have made me seriously rethink my consideration to pursue further training at [such an] institution.”* Younger male, local Doctor, #25, Hospital D *“MC rate is high. it gets demoralising. There are days I wake up not wanting to do to work.”* Younger female, local Nurse, #154, Hospital B
	*Workplace stigma (n = 5)*	Leading to a sense of disunity *“[…] Also have colleague who deliberately shun me when they know that I just finished my shift at screening centre. Is a mixed feeling. You want to fight the battle together yet not be discouraged by such acts.”* Younger female, local Allied Health Provider, #65, Hospital D
External—public and policy	*Feeling stigmatized, discriminated against by the wider community (n = 17)*	Sense of disunity was further emphasized either because healthcare workers are assumed to have or have declared having close contact with COVID-19 patients *“During the time of SARS, as a nursing personnel […] in uniform in public transportation especially in buses and mrt […] they will cursed and scolded you standing or sitting next to them. It does [happen] during this pandemic of COVID 19.”* Older female, local Nurse, #73, Hospital B *“Want to move out. But noted agents/landlords/owners prefers non healthcare tenants. Some rents seems went high.”* Middle-aged male, non-local Nurse, #19, Hospice A
	*Calls for better pay and provisions for healthcare workers (n = 16)*	National policy catering for better provision for healthcare workers, e.g., better pay and working conditions, certain privileges such as green lanes for shopping, and enabling healthcare workers seeking to travel. *“Imagine ground staffs who are so busy at work have to queue up for food together with the public who are not working due to CB, without priority lane for HCW who needs to be back at work station ASAP due to manpower stretched.”* Middle-aged female, non-local Nurse, #3, Hospital B More equity between foreign and local healthcare workers *“Monetary assistance were only available for Singaporeans yet majority of Nurses are non-Singaporeans. The effort and sacrifices they put in are not compensated in anyway because of their nationality. There is no equality in treatment.”* Middle-aged female, non-local Nurse, #3, Hospital B
	*Frustration about public apathy (n = 7)*	Caused by those observed not to be following rules and guidelines and/or being ignorant about the importance of preventative habits *“[…] it is very annoying and stressful […] many people have died, suffered & lost their livelihood [sic] die to the careless behavior of a selected few who have strange habits. This has been a needless emotional & financial & physical suffering of multitudes of innocent people worldwide.”* Older female, local Doctor, #4, Hospital A

#### 4.1.2. Heartware: Morale Boosters

While some shared that they struggled to adapt, many adopted *a stoic acceptance to fight, adjust and hold the line* (*n* = 138), characterized mostly by a sense of devotion to service, “we have to work under such conditions” and the expression or inference that “fear is for the weak”. At the absolute end of this spectrum, it was suggested that for those seen to be lacking in commitment, punitive measures should be considered.

However, far more mentioned being *motivated and inspired by strong leadership and supportive colleagues* (*n* = 67). Leadership that went the extra mile both for staff and patients when navigating uncharted territory was lauded by both local and non-local staff. Despite the risks involved in serving at the frontlines, some HCWs said they derived assurance from working with highly competent peers that they respected.

Still, more wrote of having *pride in being healthcare workers—finding a sense of purpose in one’s work* (*n* = 61). This theme cross-cut age, gender and nationality but those considered on the frontline or working with COVID-19-positive patients particularly spoke of self-reminding about their professional commitment to serve in the face of fear or, having gained clarity of purpose and job satisfaction. Whether administrative or clinical, again, particularly those on the frontline dealing with suspect or positive cases expressed being *appreciative of being thanked and supported at work* (*n* = 16). 

This was experienced through kind words and tokens of appreciation at work, inclusively distributed to all healthcare workers, as well as more tangible support such as provision of interim staff housing or restoring their ability to take leave over consecutive days. Ultimately, these positive narratives resulted in HCWs sharing that they were *able to better connect with patients* (*n* = 11), having learnt to be more empathetic, compassionate and to put others first. More broadly, gaining a greater sense of solidarity with the public (n = 19) was highlighted, particularly with reference to feeling like they are helping to fight a shared battle against a common enemy. By extension, in some cases, *pride and feeling appreciated were enhanced by public recognition and gratitude* (*n* = 7), as such, public displays of gratitude outside of work were noted.

**Table 2:** Summary of themes and sub-themes with illustrative quotes for breaking morale, hindering engagement and commitment of healthcare workers—Heartware: morale boosters.

Themes		Supporting Sub-Themes with Illustrative Quotes
Internal—Institutional	*Stoic acceptance to fight, adjust and hold the line (n = 138)*	“We have to work under such conditions” *“As healthcare workers, be it in the front line or back room, we should always adopt a mindset that such pandemic is inevitable and we have to work under such situation. This will prepare use mentally and emotionally when are placed in this condition.”* Middle-aged male, local Administrative Staff, #15, Polyclinic B “Fear is for the weak” *Well, I signed up to be a solider. I have been trained and equipped well. When it is time to go to war, I just go. Yes, it is scary. Yes, I could be killed. […] I will do my best to fight the enemy and put aside any other feeling towards it…I put myself forward to the most dangerous missions. If I die on the field, that it my fate. No hesitation. Only determination.”* Middle-aged male, non-local Doctor, #116, Hospital E
	*Motivated and inspired by strong leadership and supportive colleagues (n = 67)*	Leadership that went the extra mile both for staff and patients when navigating uncharted territory were lauded *“My nursing colleagues, general medicine and geriatric doctors, and the operations team are inspirational. I have witnessed people making personal sacrifices, working way beyond their normal hours and job scope to put in place measures to help patients coming in with COVID-19. Their dedication humbles me, inspires me and pushes me to deliver alongside them. It is an honour to work with such truly caring people. Despite their exhaustion, they still greet me with a smile […] My superheroes.”* Middle-aged female, local Administrative Staff, #88, Hospital B *“And most of all, having out Team Leader […] who selflessly devoted [herself] to ensure that we healthcare workers are safe and sound […] I am beyond grateful. Working in this kind of Pandemic is much easier if you know that someone got your back.”* Middle-aged, non-local Nurse, #194, Hospice A Derived assurance from working with highly competent peers that they respected *“Being in the front lines in the ICU and wards knowing that I am working with some or if not the best healthcare professionals gives me confidence and some peace of mind knowing that we are here to save lives.”* Younger male, non-local Allied Healthcare Provider, #10, Hospital C
	*Pride in being healthcare workers–finding a sense of purpose in one’s work (n = 61)*	Self-reminding about professional commitment to serve in the face of fear *“Being at the frontline…makes me feel at high risk for contracting it. But being reminded of my profession as a Nurse gives me the courage and strength to face it with a brave heart.”* Younger female, non-local Nurse, #8, Hospice B Clarity of purpose and job satisfaction *“There is a sense of empowerment when we start to identify what we are really meant to do. It grows out of learning what matters most to you. Once we have it, it provides direction and focus, it brings clarity and it establishes goals. It helps us achieve results and stay connected to meaningful thoughts and actions.”* Middle-aged female, non-local Nurse, #194, Hospice A
	*Appreciative of being thanked and supported at work (n = 16)*	Kind words and tokens of appreciation at work, inclusively distributed to all healthcare workers *“[…] when I receive care packages from as a healthcare worker from outside organisations […it is] an encouragement to spur us on. I felt like I am in this fight together with the Nurses and doctors, even though my role in the healthcare sector is seldom in the limelight/celebrated […] even my husband does not think of me as a healthcare worker since I do not wear a uniform, even though I come in contact and interact with the patients daily. As such, when I am recognised as one, I am thankful!”* Younger female, Administrative Staff, #8, Hospice A Thankful for tangible support, e.g., provision of interim staff housing and restoring being able to take leave over consecutive days. *“Company provided hotel to stay when the COVID 19 just started, this make [sic] me have enough time frame for [planning] where to stay. This help [sic] me avoid [a lot] of stress.”* Younger female, non-local Pharmacy Technician, #25, Polyclinic A
	*Able to better connect with patients (n = 11)*	Learnt to be more empathetic, compassionate and to and put others first *“I’ve been in a situation where my preference or values is not a priority because Safety comes first. As I ponder over [this], I thought about my residents in our Nursing Home. They say you don’t truly know how someone feels until you’ve walked a mile in their shoes. I admire their resilience, how they are capable to [adapt] in such a drastic change in their lives […]. This experience though me that we must all begin thinking outside the box, beyond our comfort zone […] beyond routine care.”* Middle-aged female, non-local Nurse, #194, Hospice A *“This has enabled me to better appreciate the difficulties that my patients experience in building boundaries and allowed me to be more compassionate towards their struggles in staying at home…This realisation has enabled me to be more understanding and empathetic towards them, allowing us to build a stronger therapeutic alliance towards change.”* Middle-aged female, local Allied Healthcare Provider, #30, Hospital A
External—public andpolicy	*Gaining a greater sense of solidarity with the public (n = 19)*	Helping to fight a shared battle against a common enemy *“#Fighting together works than fighting alone # We are the last lines of help, not the front [lines].”* Middle-aged female, local Nurse, #134, Hospital B *“We stand as One as this is our Home. If we dun [sic] fight and overcome this, who will?”* Middle-aged male, local Managerial Staff, #89, Hospital E
	*Pride and feeling appreciated by public recognition (n = 7)*	Public displays of gratitude outside of work were noted *“People now appreciated our importance and express [this] thank you. They look at our work as a dirty work previously.”* Younger female, non-local Nurse, #92, Hospital B *“Appreciation sponsorships (even if it is just a free drink) motivates us to continue caring for the public.”* Middle-aged female, local Nurse, #18, Polyclinic A

### 4.2. What Barriers and Enablers Were Experienced That Affected Operational Safety and Functioning?

#### 4.2.1. Hardware: Barriers to Operational Safety and Functioning 

The most noted area of operational safety and functioning pertained to perceptions of *sub-optimal segregation strategies within wards* (*n* = 220). Calls were made for better spaces and environmental design, especially related to having dedicated eating areas, better space management and ventilation, and smarter physical zoning of teams. Concurrently, there were also calls for better person-to-person separation, e.g., of sick people being mandated to go home, flexible working hours and split-team systems, use of digital communication and telemedicine and emphasis on not interacting over meals or after work.

The *need to improve case detection, triage and admissions criteria* (*n* = 87) through various means was highlighted. Suggestions included mass testing for staff; strategic triage of suspected cases to avoid contact with other patients and only handled by staff in full PPE; community case detection, better surveillance systems; and finding better ways of managing positive cases with mild symptoms. 

Though generally *Personal Protective Equipment (PPE)* was regarded as very well provisioned (see infrastructural enablers), some spoke of *scope for better application of this* (*n* = 54). For instance, PPE needing wider implementation, e.g., being able to change to fresh masks after lunch; mask/face shield provision for all HCWs (both clinical and non-clinical), and not just those in infection control wards. There was also mention of checks needed to ensure proper application, particularly, in “red” high-risk zones.

Relatedly, the *duress of rapid, enforced infection control measures and related staffing influx* (*n* = 47) was often mentioned. Though PPE was valued, it was also uncomfortable, damaging skin, and causing headaches and over-heating. Overtime, PPE and human endurance will need to be improved to adapt to what was at the time abrupt and stressful changes to daily practices. Immediate adaptation was also needed to “zoning” protocols—separating non-exposed and exposed groups, or segregating teams in case of cluster outbreaks, which added layers of complexity to daily life and service delivery. Stress arising from such restrictions to freedoms, i.e., through zoning of hospital and being assigned to living in staff accommodation, was notable—as was the stress arising from integrating untrained, overflow staffing.

In further comments relating to operational safety, calls were made to address unintended effects of infection control measures, in particular, mental health effects of being overworked (see morale breakers) and of patients being isolated, frightened and their wishes being overlooked. In addition, for those not working from the office anymore, the lack of resources (e.g., laptops and equipment) and support for staff coping with challenges of working from home were highlighted as an area for redress. Other areas for redress included the *need* *to enhance environmental cleaning* (*n* = 29), including frequent disinfection/wipe-down of common, high-touch areas, use of air-purifiers and sensor-activated, automated doors and minimizing use of touch-screen applications.

For a few, there was a perceived *demand for training on how to be prepared during an outbreak* (*n* = 8) including improving awareness of emergency response, infection control procedures and how to improve lifestyle and behaviors to become more adaptable. Additionally mentioned by a few as needing *stepping up was internal communication* (*n* = 7), mostly because more consistent messages were needed on the ground. More broadly, *challenges of public health communication strategy* (*n* = 27) were described, due to speed, accuracy and overload of information leading to mixed messages. There were also concerns voiced over inter-agency communication, especially over not having opinions from the ground being considered and/or implemented. 

Lastly, in a couple of instances, complaints were levelled at external parties’ (e.g., industry regulars, auditors, etc.) failure to acknowledge the unique challenges faced by healthcare workers. 

**Table 3:** Summary of themes and sub-themes with illustrative quotes on barriers to operational functioning and safety as experienced by healthcare workers—Hardware: barriers to operational functioning and safety.

Themes		Supporting Sub-Themes with Illustrative Quotes
Internal—Institutional	*Sub-optimal segregation strategies within wards (n = 220)*	Calls for better spaces and environmental design especially related to having dedicated eating areas, better space management and ventilation, and smarter physical zoning of teams *“Due to limited space, [noticed] certain Depts are still quite ‘crowded’ at times. More avail places for eating alone.”* Older female, Managerial Staff, #118, Hospital D Calls for better person-to-person separation, e.g., of sick people being mandated to go home, flexible working hours and split-team systems, use of digital communication and telemedicine, and emphasis on not interacting over meals or after work *“Some colleague mild flu and cough still come to work they think they are fine never think of others.”* Older female, local Administrative Staff, #15, Polyclinic A *“[…] Staggering work hours and work from home arrangements will be useful for [minimizing] contact between staff.”* Middle-aged female, local Allied Health, #27, Hospital D
	*Need to improve case detection, triage and admissions criteria protocols (n = 87)*	Mass testing for staff *“Maybe all Nurses that assign in COVID ward must swab first before assigning in normal ward.”* Middle-aged female, non-local Nurse, #74, Hospital B Strategic triage of suspected cases to avoid contact with other patients, and only handled by staff in full PPE *“Suspected cases of COVID patients [shouldn’t] be allowed to come to appointed clinic at all. Instead they should be seen in the isolated area. Patient should be handled […] in specific area. Less movement of the patient.”* Older female, local Nurse, #21, Hospital E Community case detection, better surveillance systems *“Having designated swab centres in the community.”* Middle-aged male, non-local Doctor, #71, Polyclinic A Better ways of managing positive cases with mild symptoms *“A lot patients do not need to be admitted to hospital honestly. Waste of time and space and resources for everyone.”* Middle-aged female, local Doctor, #2, Polyclinic B
	*Scope for better application of Personal Protective Equipment (PPE, n = 54)*	PPE needing wider implementation *“Asking front liner to wear 1 mask throughout the day. Keeping used face mask in a bad and re-use after lunch.”* Middle-aged female, local Allied Healthcare Provider, #127, Hospital B *“All healthcare staff in the nation should be already mask-fitted with N95 in advance and not to be done at last minute.”* Older female, local Nurse, #9, Polyclinic A *“As a staff involved in entrance screening, no face shield given, I felt we had to just count our blessings of not getting infected.”* Older female, local Managerial Staff, #100, Hospital D Checks needed to ensure proper application, particularly in “red” high-risk zones *“Stress happened when I saw some Healthcare workers on non-health care staff did not remove the PPE in correct sequence.”* Middle-aged female, non-local Nurse, #35, Hospital C
	*Duress of rapid, enforced infection control measures and relaxed staffing influx (n = 47)*	PPE is uncomfortable, damaging skin, and causing headaches and over-heating *“The N95 masks we wear, they are tight and uncomfortable but necessary. They leave marks on our faces and increase out breathing.”* Younger female, local Nurse, #154, Hospital B *“On my First Day of deployment [to migrant worker dormitories in tentage areas]…I experienced a terrible headache from wearing the face shield […]. Wearing a full set of PPE under such conditions was unbearable. One of my colleagues vomited from the extreme heat.”* Middle-aged female, local Allied Healthcare Provider, #4, Polyclinic B Stress arising from restrictions to freedoms, i.e., through zoning of hospital and being assigned to living in staff accommodation *“You […] caused my stress by zoning us! We were staying with our family, whom we lived for years and you […] zoned us. Coping with new housemates? use your big brains next time.”* Middle-aged male, non-local Nurse, #118, Hospice A Stress arising from integrating untrained, overflow staffing *“They have cause chaos by appointing non-lab people to run screening and testing.”* Older male, non-local Doctor, #7, Hospital D Calls were made to address unintended effects of infection control measures, in particular mental health effects of staff being overworked and of patients being isolated, frightened and their wishes being overlooked *“This period has been especially tough […] for nursing home [residents…]. The elderly were more socially isolated during this period. We saw residents deteriorating with poor oral intake. We saw nursing homes transferring patients to hospital as they are worried of patient having COVID-19 despite patient’s wishes not to go hospital.”* Middle-aged female, local Doctor, #29, Hospital D Lack of resources (e.g., laptops and equipment) and support for staff coping with challenges of working from home was highlighted as an area for redress *“Can we have just a laptop for work and access to internet. There should be better control by the IT side.”* Older female, local Administrative Staff, #15, Polyclinic A
	*Need to enhance environmental cleaning (n = 29)*	Frequent disinfection/wipe-down of common, high-touch areas, use of air-purifiers *“Daily cleaning [needed] on highly contact touching areas.”* Older female, local Nurse, #3, Hospice B Sensor-activated, automated doors and minimize use of touch-screen applications “Would be good if there were more sensor doors. Or at least fix the sensor***to be more sensitive to the staff access tag.” Younger female, local Allied Healthcare Provider, #49, Hospital B
	*Demand for training on how to be prepared during an outbreak (n = 8)*	Including improving awareness of emergency responses, infection control procedures, and how to improve lifestyle and behaviors to become more adaptable *“Sufficiently trained staff to ensure compliance and awareness infection control standards especially the cleaning team.”* Older female, local Nurse, #9, Polyclinic A
	*Stepping up internal communication (n = 7)*	Consistent messages needed on the ground *“[…] there were [a lot] of different jargons that were created for COVID and different people have different definitions of them. it confuses the ground and there were [sic] no one to clarify with especially the initial phase as we were clueless.”* Middle-aged female, local Nurse, #10, Hospital B
External—public andpolicy	*Challenges of public health communication strategy (n = 27)*	Speed, accuracy, and overload of information, leading to mixed messages *“Not coping, send help. Policies are ever changing Politicians are flipping their stance It is so confusing.”* Middle-aged male, local Doctor #26, Hospital D Concerns voiced over inter-agency communication, especially over not having opinions from the ground heard, and opinions recognized *“I beseech minister [emphasis our own] secure the return of CDC 1 to be used for its original purpose.”* Older female, local Doctor, #88, Hospital D
	*External parties’ failure to acknowledge unique challenges faced by healthcare workers (n = 2)*	In particular, industry regulars/auditors *“Auditors were not sympathetic […] when explanations were given with regards to manpower shortage etc. That was very stressful, as many of my staff wanted to resign […]. we had to do counselling to ensure they stay. […] Auditors should be trained well to handle staff during this period when everyone is working really hard.”* Older female, local Nurse #7, Hospice A

#### 4.2.2. Hardware: Enablers to Operational Safety and Functioning 

With respect to *Personal Protective Equipment (PPE),* many spoke of *timely and well-planned provisions for these front-liners* (*n* = 41). Timely access was readily acknowledged by these HCWs, and gratitude often expressed. This was even to the point that these groups were seen as less at risk of infection in general, compared to non-frontline facing potentially suspect cases with no protection. Besides PPE, which was an area of preparedness that had laid in readiness since SARS, HCWs also spoke up *in praise of other fast-enacted adaptive strategies that worked* (*n* = 25). 

These included addressing manpower shortages with staff on loan which worked well if these were well matched to skills that were required or simply an influx of less skilled staff, such as cleaning crew ramp-ups. Some were especially grateful for the quick delivery of infection control support, e.g., swabbing and washing/provision of scrubs for front-liners within the first month or so of community lockdown. Others mentioned using ingenuity and innovation, improvising with what was available to improve infection control, such as making “swab shields’ to protect themselves from patients coughing or vomiting while doing the COVID-19 swab. There were also some accounts of very positive experiences of those transitioning to working from home.

In addition, external factors such as expressions granting *political legitimacy to mitigation initiatives and speaking of trust gained through inter-agency collaborations* (*n* = 38) supported continuation of operations and safety protocols. For instance, demonstrable efficiency in healthcare systems, evidenced by good outcomes, was referenced—as was emphasis on the importance of public willingness to comply with community restriction. More specifically, the benefits of good inter-agency communication and collaboration were highlighted. Lastly, in long-practicing HCWs, we observed *appreciation of being able to rely on lessons learnt from previous outbreaks* (*n* = 18), especially legacy improvements from SARS.

**Table 4:** Summary of themes and sub-themes with illustrative quotes on enablers to operational functioning and safety as experienced by healthcare workers—Hardware: enabling operational functioning and safety.

Themes		Supporting Sub-Themes with Illustrative Quotes
Internal—Institutional	*Timely and well-planned-for provision of Personal Protective Equipment (PPE) for front-liners (n = 41)*	Timely access was readily acknowledged by these healthcare workers, and gratitude often expressed *“Always felt protected by the PPE given, Staff always provided with good PPE. So this really helped our stress levels. Extremely thankful.”* Middle-aged male, local Doctor, #22, Hospital B *“I feel blessed. I have proper PPE and a healthy body to take care of the patients.”* Middle-aged female, non-local Nurse, #6, Polyclinic B Even to the point that these groups were seen as less at risk of infection in general, compared to non-frontliners facing potentially suspect cases with no protection *“Actually, I preferred to care [for] patients with COVID 19 because [I] felt more safe [sic]. I can wear the full PPE to protect myself rather than taking care of patient that later will have symptoms of COVID 19.”* Middle-aged female, non-local Nurse, #161, Hospital B
	*In praise of other fast-enacted adaptive strategies that worked (n = 25)*	Addressing manpower shortages with staff on loan *“[…everything is ramped up…] environmental cleanliness has been given so much emphasis. [Ward] ramped up its resources rapidly, requests for additional support streamed in over the CNY break.”* Middle-aged female, local Managerial Staff, #36, Hospital D Quick delivery of infection control support, e.g., swabbing and washing/provision of scrubs for frontliners within the first month or so of community lockdown *“[…] I am really glad that by end May, our staff policy changed and now swab everyone who presents with [respiratory infection] regardless of their working location.”* Younger female, local Allied Health Provider, #90, Hospital D Ingenuity and innovation, adapting what was available for better infection control, such as “swab shields” *“We have created a “swab shield” in screening centre […], (a clear plastic material mounted on portable drip stand) to be use in order to protect ourselves from patients who might cough or vomit while we are doing the COVID swab.”* Middle-aged female, local Nurse, #138, Hospital D Positive experiences of those transitioning to work from home *“[…] I am grateful that my department had put in work from home measure when dorscon was changed to orange. During this period which we are working from home, work can still be done and in fact more efficiently than in office, where there are more distractions. Having COVID also gives us the opportunity to adopt working from home and it is still viable to continue this practise even after vaccine has been found for COVID.”* Middle-aged female, local Administrative Staff, #11, Polyclinic A
External—public andpolicy	*Political legitimacy to mitigation initiatives and trust gained through inter-agency collaborations (n = 38)*	Demonstrable efficiency in healthcare systems—evidenced by good outcomes *“Initially when the pandemic started, i was really worried […] But subsequently all the implementations done by government really surprised me, the welfare of FW were well protected! […] the numbers of community COVID cases had a drastic drop these few days. That […] means all the measures implanted are working well.”* Young female, non-local Nurse, #49, Polyclinic A Emphasis on the importance of public willingness to comply with community restriction *“COVID-19 is real and instead of complaining; I chose to follow and comply strictly what has been implemented by the Sg Govt. As a Healthcare personnel, I often educate my family members to strictly comply to the measures. Also advised them to refer to MOH website for update rather than looking at untrue articles circulating online.”* Middle-aged female, local Nurse, #104, Hospice A The benefits of good inter-agency communication and collaboration were highlighted *“We had a contact case in our nursing home […]. It was very scary. But I was very impressed with [inter-agency response]. Within 2.5 hrs, the team was on site and within 5 hour, swabbing exercises were [performed for] all our staff.”* Older female, local Nurse Clinician, #107, Hospice A
	*Appreciation of being able to rely on lessons learnt from previous outbreaks (n = 18)*	Especially legacy improvements from SARS *“I have been helping out the mass [swab] team. We have been travelling to different areas, but not once I felt afraid. The reason because ever since after SARS happened in Singapore I felt that our Singapore medical team have been more equipped with greater knowledge and precautions measures for the staff and public. All the training I been taught has groom me to be stronger to face such pandemic.”* Middle-aged female, local Administrative Staff, #43, Polyclinic A

## 5. Discussion

### 5.1. Interpretation for Theory and Practice 

**Figure 1:**
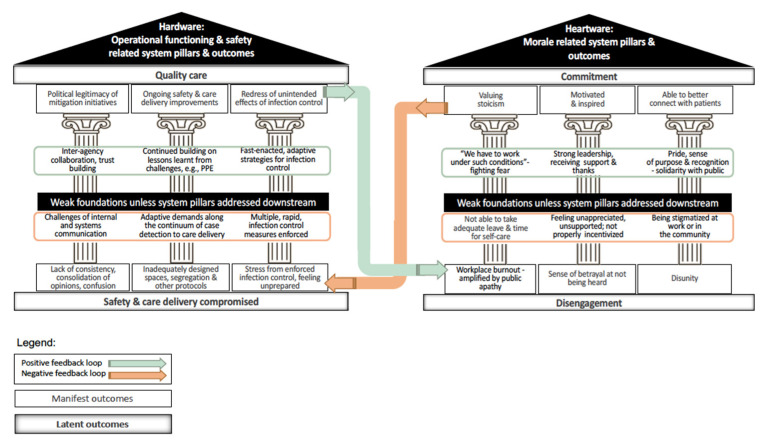
Systems model for Adaptive Pandemic Response addressing healthcare worker workplace burnout.

The current study seeks to account for the voices of HCWs [29], building on their perspectives relating to operational functioning and safety or Hardware, as well as their experiences of what shapes Heartware and underpins morale. This cross-cutting set of analyses, informed by systems thinking [30], integrates internal/institutional and external/public-policy accounts as distilled in Figure 1. 

The resultant systems model of Adaptive Pandemic Response underscores how addressing HCW workplace burnout will be central for COVID-19 healthcare provision to pivot from a reactive “wartime” response to an endemic or even intermittent “peacetime” scenario, according to the typology suggested by Singh et al. (2017) [18]. Mass vaccine rollouts are unfolding at full throttle in many countries, including Singapore, alongside plans to adopt differentiated policies that distinguish between the vaccinated versus the unvaccinated. As such, though anticipated to cause less disease severity, COVID-19 will continue to surge [31].

Adopting a stoic approach [32] can compromise patient safety and quality of care. This response is necessary when reacting in the eye of the storm; indeed, this was the biggest morale booster in the height of the first wave in Singapore. Many HCWs saw themselves as soldiers, ready for waging a war. However, this stance can wane, especially in the face of enforced infection control measures and the ongoing need for fast-enacted, adaptive strategies. Valuing stoicism will only remain a positive tool if the unintended effects of dealing with the duress of multiple, rapidly unfolding infection control measures are also, ultimately, redressed or tempered. 

Our data suggest that workforces that are motivated and inspired - mostly by strong leadership, protected from workplace burnout and other cumulative stresses, and given the recognition, pride and sense of purpose and solidarity with the public – will, be altogether more committed; likely, they will also connect better with patients. Furthermore, they will be more able to build on lessons learnt and continue to monitor and report challenges, closely collaborating across ministries and healthcare agencies, all of which were demonstrated to be well-established pillars of the strong adaptive systems-level response to the pandemic in Singapore.

### 5.2. Strengths and Limitations 

The current analysis is strengthened by a breadth of data. Though data may have lacked some depth, it was collected in “real time” across a range of healthcare institutions during a critical phase on systems response to the first wave of the pandemic. The survey, e-diary responses allowed us to collect data from a group that was at the time difficult to access. On the other hand, not everyone would have been comfortable sharing intimate experiences via a survey link received though their work-email accounts. This method of data collection will, therefore, have excluded the more suspicious. 

Another consideration is that due to the volume of data coding required, this was divided up between the analysts and we did not undertake formal, blinded, double coding. However, we did apply the proposed Hard / Heartware framework and shared at least 30% of our coding labels as tagged to data, agreeing on their meanings. A series of meetings were held, where we reached a consensus on joining and collapsing the themes and sub-themes across each person’s bundle of data.

In addition, our findings are broadly consistent with a recent systematic review [33], where, for example, burnout was identified as an overarching, cross-cutting theme. The current study is nevertheless unique in exploring a highly adaptive system that was demonstrated to have reacted very successfully during the crisis itself; it also adds a theoretical formulation and mapping of findings for future development and testing to help guide planning and policy.

## 6. Conclusions

With much attention and resources being devoted to ensuring health systems are well equipped with the necessary Hardware, more can be done to address Heartware. The current research suggests an urgent need to increase the size of the healthcare workforce, offer better pay and provisions and foster an employee-centric culture where leave can be taken and self-care promoted in the longer term. 

As countries strive to contain current variants of COVID-19 while moving towards endemicity, the results and observations of this work can be used to inform global public health policy and future pandemic planning and preparedness.

## Data Availability

The datasets used during the current study are not available due to the sensitive nature of this study and to preserve anonymity as per the study team’s commitment during institutional ethical review.

## Supplementary Materials

The following supporting information can be downloaded at: https://www.mdpi.com/article/10.3390/ijerph19042477/s1, Box S1: Questions eliciting e-diary entries; Table S1: Characteristics of participants responders and non-responders for e-diary data, *n* = 1209; Table S2: Characteristics of survey participants according to institutional affiliation (blinded), *n* = 1209.
