# Supplementary material for: “Well, I Signed Up to Be a Soldier; I Have Been Trained and Equipped Well”: Exploring Healthcare Workers’ Experiences during COVID-19 Organizational Changes in Singapore, from the First Wave to the Path towards Endemicity
Source: Int J Environ Res Public Health. 2022 Feb 21;19(4):2477. doi: 10.3390/ijerph19042477 (PMC8878310; doi:10.3390/ijerph19042477)
Supplement: Supplementary file 1 [file ijerph-19-02477-s001.zip › ijerph-1540024-supplementary.pdf]

Supplementary Box S1: Questions eliciting e-dairy entries

We would love to hear your stories about how you have been coping with the psychological stress of being at the frontlines. Please share with us a snapshot of the stress you face and what you do to cope with it. This can be in the form of a journal or diary entry, an audio recording, a photo with a brief description, or artistic expression like poetry or drawing. You can upload your entry anonymously below. This section is entirely voluntary and you will remain anonymous.

Q1: Enter journal or diary entry here

Q2: Upload photo with brief description or audio recording here

Q3: Upload photo of poetry or drawing here

Supplementary Table S1: Characteristics of participants responders and non-responders for e-diary data, based on grand total n=3616 who completed at least one survey item

		Total N=3616† Counts (%)	Total respondents for e-dairy N=663 Counts (%)	Total non-respondents for e-dairy N=2953 Counts (%)	*p*-value
Age	21yrs. to 30 yrs.	1038 (28.7)	205 (30.9)	833 (28.2)	0.344
	31 yrs. to 50 yrs.	2079 (57.5)	374 (56.4)	1705 (57.7)	
	Above 51 yrs.	494 (13.7)	84 (12.7)	410 (3.9)	
Gender	Female	2700 (74.7)	507 (76.5)	2193 (74.3)	0.258
	Male	916 (25.3)	156 (23.5)	760 (25.7)	
Nationality	Local	1694 (46.9)	282 (42.5)	1412 (47.8)	0.016*
	Non-local	1922 (53.2)	381 (57.5)	1541 (52.2)	
Marital Status	Single	1564 (43.1)	298 (44.9)	1266 (42.9)	0.286
	Married	1911 (52.9)	343 (51.7)	1568 (53.1)	
	Divorced/Widowed	155 (4.2)	22 (3.3)	133 (4.5)	
Accommodation	Alone	214 (5.9)	53 (8.0)	161 (5.5)	0.001**
	Family	2183 (60.4)	366 (55.2)	1817 (61.5)	
	Hostel	128 (3.5)	18 (2.7)	110 (3.7)	
	Rental room/apartment	1080 (29.9)	226 (34.1)	854 (28.9)	
Years of Experience	0 to 10 yrs.	2029 (56.1)	362 (54.6)	1667(56.5)	0.024*
	11 to 20 yrs.	1050 (29)	185 (27.9)	865 (29.3)	
	21 to 30 yrs.	294 (8.1)	73 (11.0)	221 (7.5)	
	More than 30 yrs.	210 (5.8)	43 (6.5)	167 (5.7)	
Cadre	Doctor	305 (8.4)	69 (10.4)	236 (8.0)	
	Nurse	1870 (51.7)	327 (49.3)	1543 (52.7)	0.184
	Allied health	677 (18.7)	128 (19.3)	549 (18.8)	
	Administrative etc.	739 (20.4)	139 (21.0)	600 (20.5)	
COVID-19 Daily Exposure	Yes	404 (11.2)	111 (16.7)	293 (9.9)	< 0.001***
	No	3212 (88.8)	552 (83.3)	2660 (90.1)	

†*Reproduced with permission from Chan et al, 2021* [27], *based on* n=3616 who completed at least one survey item
Note. *p <.05; **p <.01; ***p <.001

Supplementary Table S2: Characteristics of survey participants according to institutional affiliation (blinded), n=663

		Total N=663 Count (%)	Hospital **A** N=117 Counts(%)	Hospital **B** N=111 Counts(%)	Hospital **C** N=38 Counts(%)	Hospital **D** N=126 Counts(%)	Hospital **E** N=25 Counts(%)	Hospice **A** N=178 Counts(%)	Hospice **B** N=8 Counts(%)	Polyclinic **A** N=44 Counts(%)	Polyclinic **B** N=16 Counts(%)	*p*-value
Age	21yrs. to 30 yrs.	205 (30.9)	26 (22.2)	11 (28.9)	11 (28.9)	32 (25.4)	13 (52.0)	3 (37.5)	68 (38.2)	13 (29.5)	2 (12.5)	0.07
	31 yrs. to 50 yrs.	374 (56.4)	71 (60.7)	26 (68.4)	26 (68.4)	76 (60.3)	11 (44.0)	4 (50.0)	90 (50.6)	24 (54.5)	12 (75.0)	
	Above 51 yrs.	84 (12.7)	20 (17.1)	1 (2.7)	1 (2.7)	18 (14.3)	1 (4.0)	1 (12.5)	20 (11.2)	7 (15.9)	2 (12.5)	
Gender	Female	507 (76.5)	80 (68.4)	32 (84.2)	32 (84.2)	101 (80.2)	16 (64.0)	6 (75.0)	139 (78.1)	36 (81.8)	10 (62.5)	0.17
	Male	156 (23.5)	37 (31.6)	6 (15.8)	6 (15.8)	25 (19.8)	9 (36.0)	2 (25.0)	39 (21.9)	8 (18.2)	6 (37.5)	
Nationality	Local	283 (42.7)	56 (47.9)	19 (50.0)	19 (50.0)	75 (59.5)	9 (36.0)	3 (37.5)	25 (14.0)	27 (61.4)	13 (81.2)	$<0.001^{***}$
	Non-local	380 (57.3)	61 (52.1)	19 (50.0)	19 (50.0)	51 (40.5)	16 (64.0)	5 (62.5)	153 (86.0)	17 (38.6)	3 (18.8)	
Marital Status	Single	298 (44.9)	40 (34.2)	14 (36.8)	14 (36.8)	47 (37.3)	14 (56.0)	7 (87.5)	108 (60.7)	15 (34.1)	3 (18.8)	$<0.001^{***}$
	Married	343 (51.7)	72 (61.5)	21 (55.3)	21 (55.3)	75 (59.5)	11 (44.0)	1 (12.5)	64 (36.0)	27 (61.4)	13 (81.2)	
	Divorced/Separated	14 (2.1)	3 (2.6)	3 (7.9)	3 (7.9)	3 (2.4)	0 (0)	0 (0.0)	1 (0.6)	2 (4.5)	0 (0)	
	Widowed	8 (1.2)	2 (1.7)	0 (0)	0 (0)	1 (0.8)	0 (0)	0 (0)	5 (2.8)	0 (0)	0 (0)	
Accommodation	Alone	53 (8.0)	14 (12.0)	6 (15.8)	6 (15.8)	10 (7.9)	0 (0)	1 (12.5)	11 (6.2)	4 (9.1)	0 (0)	$<0.001^{***}$
	Family	367 (55.4)	72 (61.5)	23 (60.5)	23 (60.5)	99 (78.6)	14 (56.0)	3 (37.5)	37 (20.8)	32 (72.7)	16 (100)	
	Hostel	18 (2.7)	0 (0)	0 (0)	0 (0)	0 (0)	1 (4.0)	0 (0)	17 (9.6)	0 (0)	0 (0)	
	Rental room/ apartment	225 (33.9)	31 (26.5)	9 (23.7)	9 (23.7)	17 (13.5)	10 (40.0)	4 (50.0)	113 (63.5)	8 (18.2)	0 (0)	
	0 to 10 yrs.	362 (54.6)	52 (44.4)	21 (55.3)	21 (55.3)	59 (46.8)	17 (68.0)	3 (37.5)	120 (67.4)	23 (52.3)	5 (31.2)	0.03^{*}

Years of Experience	11 to 20 yrs.	185 (27.9)	40 (34.2)	13 (34.2)	13 (34.2)	44 (34.9)	4 (16.0)	2 (25.0)	34 (19.1)	12 (27.3)	6 (37.5)	
	21 to 30 yrs.	73 (11.0)	15 (12.8)	4 (10.5)	4 (10.5)	12 (9.5)	4 (16.0)	2 (25.0)	13 (7.3)	7 (15.9)	3 (18.8)	
	More than 30 yrs.	40 (6.0)	9 (7.7)	0 (0)	0 (0)	11 (8.7)	0 (0)	1 (12.5)	9 (5.1)	2 (4.5)	2 (12.5)	
	Missing	*3 (0.5)*	*1 (0.9)*	*0 (0)*	*0 (0)*	*0 (0)*	*0 (0)*	*0 (0)*	*2 (1.1)*	*0 (0)*	*0 (0)*	
Cadre	Doctor	69 (10.4)	5 (4.3)	11 (10.0)	1 (3.6)	32 (25.4)	5 (20.0)	0 (0)	1 (12.5)	10 (22.7)	4 (25.0)	
	Nurse	327 (49.3)	74 (63.2)	55 (49.5)	9 (32.1)	35 (27.8)	17 (68.0)	109 (61.2)	6 (75.0)	9 (20.5)	3 (18.8)	
	Allied health	128 (19.3)	31 (26.5)	22 (19.8)	7 (25.0)	35 (27.8)	2 (8.0)	20 (11.2)	0 (0)	8 (18.2)	3 (18.8)	<0.001***
	Administrative etc.	139 (21.0)	7 (6.0)	23 (20.7)	11 (39.3)	24 (19.0)	1 (4.0)	49 (27.6)	1 (12.5)	17 (38.6)	6 (37.4)	
COVID-19 Exposure	Yes	229 (34.5)	20 (17.1)	20 (52.6)	20 (52.6)	61 (48.4)	17 (68.0)	3 (37.5)	14 (7.9)	24 (54.5)	6 (37.5)	<0.001***
	No	434 (65.5)	97 (82.9)	18 (47.4)	18 (47.4)	65 (51.6)	8 (32.0)	5 (62.5)	164 (92.1)	20 (45.5)	10 (62.5)	

Note. **p* <.05; ****p* <.001
